# Elevated C-reactive protein is associated with suicide attempts in youth with bipolar disorder

**DOI:** 10.1017/S0033291726103948

**Published:** 2026-04-13

**Authors:** Dharmayu Desai, Mikaela K. Dimick, Kody G. Kennedy, Alysha A. Sultan, Megan Mio, Benjamin I. Goldstein

**Affiliations:** 1 Centre for Youth Bipolar Disorder, Centre for Addiction and Mental Health, Toronto, Canada

**Keywords:** bipolar disorder, C-reactive protein, suicide, youth

## Abstract

**Background:**

C-reactive protein (CRP) has been studied in relation to bipolar disorder (BD) and suicidality independently. Although suicide risk is elevated in youth with BD, little is known about the association of CRP with suicidality in this population.

**Methods:**

211 youth participated, including 23 BD with lifetime suicide attempts (BD_SA_), 45 BD with lifetime non-suicidal self-injury (NSSI; BD_NSSI_), 39 BD without lifetime suicide attempt or NSSI (BD_No-SA/NSSI_), and 104 healthy controls (HC). Suicide attempts and NSSI were assessed systematically. Fasting blood samples yielded CRP levels. Primary analyses controlled for age, sex, and body mass index percentile.

**Results:**

CRP levels differed across groups (*F*_3,204_ = 3.40, *p* = 0.02, *η_p_* ^2^ = 0.05). In post hoc analyses, CRP levels were significantly higher among BD_SA_ (3.44 ± 6.42 mg/L) vs HC (0.81 ± 0.90 mg/L; *p* < 0.01) and BD_No-SA/NSSI_ (1.42 ± 3.31 mg/L; *p* = 0.01) groups; however, no difference was seen with the BD_NSSI_ group (1.83 ± 2.22 mg/L; *p = 0.12*). Between-group differences in CRP levels persisted in independent sensitivity analyses controlling for current mood symptoms, lifetime mania score, lifetime smoking, and medications, but not with lifetime depression score.

**Conclusions:**

Suicide attempts among youth with BD are associated with elevated CRP. Given accessibility of CRP testing, the present findings have potential clinical implications. Larger, longitudinal studies with repeated measures are needed to examine time-varying associations between CRP and suicide risk among youth with BD.

## Introduction

Suicide is the second leading cause of death in youth in Canada and the United States (Government of Canada, 2023). Suicide risk factors in youth include prior suicidal behavior, psychiatric disorders, and environmental stressors (Steele, Thrower, Noroian, & Saleh, 2018). Bipolar disorder (BD), a severe psychiatric condition characterized by manic and/or hypomanic episodes generally alternating with depressive episodes, confers a markedly increased risk for suicide (McIntyre, Berk, Brietzke, et al., 2020). Moreover, about a quarter of individuals with BD attempt suicide at least once in their lifetime (Schaffer, Isometsa, Tondo, et al., 2015). Similar rates of deaths by suicide and attempts are seen in studies specifically examining early-onset BD (Goldstein, Ha, Axelson, et al., 2012; Serra, De Crescenzo, Maisto, et al., 2022), with a longitudinal analysis citing persistent depression, substance use disorder, and family history of suicide as additional risk factors (Goldstein, Ha, Axelson, et al., 2012). Given that BD commonly onsets during adolescence (Bolton et al., 2020) and suicide is a leading cause of death in youth (Government of Canada, 2023), it is critical to examine risk factors for suicide in youth with BD. Of note, longitudinal studies in youth with major depressive disorder have found that non-suicidal self-injury (NSSI; i.e. self-harm without intention to die) and suicide attempt (SA; i.e. self-harm with intention to die) present equivalent risk of future suicide attempt (Wilkinson et al., 2011).

In addition to psychiatric risk factors for suicide, it is important to integrate biological risk factors. Biological processes implicated in suicide risk include genome and epigenomic measures, structural brain differences, hypothalamic–pituitary–adrenal axis (HPA) dysregulation, peripheral and neuroinflammatory pathways, and serotonergic abnormalities (Abou Chahla et al., 2023). Among these putative mechanisms, inflammation is especially relevant given its strong, replicated associations with both BD and suicide (Abou Chahla et al., 2023; Goldstein, Kemp, Soczynska, & McIntyre, 2009; Modabbernia, Taslimi, Brietzke, & Ashrafi, 2013). Whereas most inflammatory markers that have been reported in prior studies require research-specific assays, C-reactive protein (CRP) is a commonly measured inflammatory marker that is inexpensive and widely available at clinical laboratories (Pepys & Hirschfield, 2003). Release of CRP from the liver is stimulated by cytokines (e.g. interleukin-6 (IL-6), interleukin-1, and tumor necrosis factor-α (TNF) and is hypothesized to regulate acute inflammatory responses by facilitating phagocytosis (Sproston & Ashworth, 2018). Meta-analyses show higher levels of CRP in adults with psychiatric disorders and a history of suicidal ideation and/or behaviors compared to the control group (Miola, Dal Porto, Tadmor, et al., 2021). This relationship remained true when constraining analyses to a sample of adults with BD (Ducasse, Jaussent, Guillaume, et al., 2015; Miola, Dal Porto, Tadmor, et al., 2021). Compared to healthy controls (HC), adults with BD showcase elevated CRP levels during euthymia and depression, with the highest levels observed during mania, as demonstrated in meta-analytic findings (Fernandes, Steiner, Molendijk, et al., 2016). Moreover, current literature demonstrates higher CRP levels in youth with BD compared to HCs and positive correlations between CRP levels and mood symptom severity in individuals with BD (Munkholm et al., 2024; Zou et al., 2022a).

Taken together, elevated CRP is associated with suicide attempts, BD, and the presence of suicide attempts among adults with BD specifically. However, studies have yet to examine the association of CRP with suicide risk (i.e. suicide attempts and NSSI) in youth with BD. In a subset of the current sample, our group has investigated associations of CRP with BD symptomatic status in youth cross-sectionally and prospectively (Karthikeyan, Dimick, Fiksenbaum, et al., 2022; Zou et al., 2022a). The aforementioned studies demonstrated elevated CRP in symptomatic youth with BD compared to HCs and greater CRP levels predicting increased time to recovery from the initial symptomatic episode (Karthikeyan, Dimick, Fiksenbaum, et al., 2022; Zou et al., 2022a). Given the early onset of BD and the high risk of suicide attempts and NSSI in youth with BD, along with prior evidence that inflammation is relevant to youth BD, we set out to examine potential relationships of CRP with suicide attempts and NSSI in this population. Notably, current literature highlights lower adverse childhood experiences, fewer depressive symptoms, greater self-esteem, and parental support in youth with NSSI compared to youth with lifetime suicide attempt (Brausch & Gutierrez, 2009; Masi, Lupetti, D’Acunto, et al., 2021). These differences in risk factors for NSSI and suicide attempt in youth may relate in part to the association of CRP levels with the type of self-harm. For example, whereas higher CRP levels are associated with increased risk of lifetime suicide attempts (Courtet, Jaussent, Genty, et al., 2015), two youth studies found that CRP levels were not associated with NSSI (Bai, Asarnow, Babeva, & Irwin, 2024; Kindler et al., 2022). We therefore hypothesized that CRP levels would be highest among youth with BD and a history of suicide attempt, followed by youth with BD and a history of NSSI, youth with BD with no prior suicide attempt or NSSI, and HC.

## Methods

### Participants

Participants included English-speaking youth ranging between 13 and 21 years of age. Youth who met diagnostic criteria for BD-I (bipolar I disorder), BD-II (bipolar II disorder), or BD-NOS (Not Otherwise Specified; akin to Other Specified Bipolar and Related Disorder) were recruited from a tertiary subspecialty clinic in Toronto, Canada. Youth were recruited for four different study protocols (including biomarker and neuroimaging studies) that used the same interview measures, CRP measurement, and protocol described in this study (REB 032–2012, 347–2011, 409–2013, and 405–2014). The Kiddie Schedule for Affective Disorders and Schizophrenia for School Age Children, Present and Lifetime version (K-SADS-PL) semi-structured diagnostic interview was administered to all participants to determine lifetime history of psychiatric diagnoses (Kaufman, Birmaher, Brent, et al., 1997). HC youth were recruited through advertisements within the local community. Prior to involvement in any study procedures, written informed consent was obtained from all participants in addition their parents/guardians. Participants from all groups were excluded if they were unable to provide informed consent or used anti-inflammatory medication, or indicated an infectious disease within the past 14 days. Additionally, participants with pre-existing cardiac, inflammatory, or autoimmune conditions were excluded. HC participants were screened for the absence of mood or psychiatric disorders, psychotic disorders, exposure to psychiatric medication within the past three months, family history of BD in first or second degree, or recent alcohol/drug dependence. A total of 107 youth with BD and 104 HC youth were eligible for participation. All study protocols were approved by the local research ethics board.

### Psychiatric measures

To confirm clinical diagnoses of BD-I and BD-II, the *Diagnostic and Statistical Manual of Mental Disorders* (*DSM-IV*) was utilized as the recruitment for the study began in 2012, prior to the publication of the K-SADS-PL corresponding to the *Diagnostic and Statistical Manual of Mental Disorders, 5th Edition (DSM-5)* (*Kaufman, Birmaher, Brent, et al., 1997*). Research staff with at least a bachelor’s or master’s degree conducted interviews to collect information using the aforementioned measures, with diagnoses subsequently confirmed by a child-adolescent psychiatrist. Diagnosis for BD-NOS was confirmed using the *DSM-5* symptom count requirements, determining duration of symptoms and number of hypomanic days as operationalized and outlined in the Course of Bipolar Youth (COBY) study (Birmaher, Axelson, Strober, et al., 2006). The age when participants first experienced hypomania or mania was defined as the age of onset of BD. Severity of mood symptoms for the current mood episode (worst week in the past month) and most severe lifetime episode were assessed using participant scores on the KSADS Depression Rating Scale (DRS) and Mania Rating Scale (MRS) (Axelson, Birmaher, Brent, et al., 2003; Chambers, Puig-Antich, Hirsch, et al., 1985). The Family History Screen Interview was utilized to assess family psychiatric history in first- and second-degree relatives (Weissman et al., 2000), while the K-SADS-PL interview gathered data on medication, substance use, and lifetime cigarette smoking. Research Electronic Data Capture (REDCap) tools were utilized to collect and organize data, with record audit trails being recorded regarding study data manipulation and export (Harris et al., 2009; Harris, Taylor, Minor, et al., 2019).

### Suicide risk assessment

The Adolescent Longitudinal Interval Follow-Up Evaluation (A-LIFE) Self-Injurious/Suicidal Behavior Scale was utilized to assess past suicidal attempts or NSSI, with additional information collected regarding method, intent, and medical threat (Keller, Lavori, Friedman, et al., 1987). A score of greater than or equal to 3 on both medical threat (‘mild’) and level of stated intent (‘definite but still ambivalent’) was considered a suicide attempt. Scores less than 3 within medical threat and/or intent were classified as NSSI. Individuals with no self-harm included participants with no history of suicide attempt and NSSI. Information was collected directly from youth, informants, parent/guardian reports, and available medical records. Sources of information were cross-referenced to confirm the history of suicide attempt. A detailed description of the A-LIFE Self-Injurious/Suicidal Behavior and PSR scales can be found in Supplemental Table 1.

### Anthropometric measures

Physical development was evaluated as Tanner Stage using the Pubertal Developmental Scale (Petersen, Crockett, Richards, & Boxer, 1988). A digital scale and wall-mounted stadiometer were used to measure participant weight and height twice, with the average obtained for accuracy purposes (Krebs et al., 2007). Height and weight were measured twice to calculate BMI using standardized procedures (Krebs et al., 2007). Weight was adjusted for clothing differences, specifically by subtracting 1.3 kg for participants in long pants and a long-sleeved shirt, 1.1 kg for short pants or a short-sleeved shirt, and 0.9 kg for short pants and short-sleeved shirts. A third measurement was conducted when there was a difference of >1 cm in height or > 0.3 kg in weight between the first two measurements (Krebs et al., 2007). BMI percentile is the measure suggested by the CDC for youth, as natural growth in height and weight are not accounted for in absolute BMI measurements (Harrington et al., 2012). According to CDC growth charts, for participants aged from 13 years to 19 years and 11 months, BMI percentile was calculated using age and sex-specific reference data. As CDC reference data are not available beyond age 20, the age 19 BMI percentile cutoffs were applied to participants aged 20–21 (Centers for Disease Control and Prevention, 2025).

### CRP assay

Participants were asked to fast for 10 hours prior to blood sample collection via antecubital venipuncture. Additionally, participants were instructed to abstain from tobacco, alcohol, and illicit drug use for 24 hours before their study visit. Trained hospital laboratory staff, blind to participant grouping, measured CRP levels in serum with a minimum detection level of 0.2 mg/L using the Tina-Quant C-Reactive Protein (CRPL3) Gen.3 turbidimetric immunoassay on a Roche Cobas® 702 analyzer (Roche Diagnostics, IN, USA). Values reported as <0.2 mg/L were entered as 0.2 mg/L. CRP was measured at the hospital clinical laboratory in real time and was not stored in a freezer prior to analysis. As a result, information regarding coefficients of variation is not available.

### Selection of covariates

The covariates were selected based on prior literature and associations with CRP and to maintain consistency with other studies by our group. Age and sex were included as they account for significant biological and demographic differences. As Tanner’s stage is correlated with age, it was not included in the analysis. CRP has been associated with mood symptom burden in youth with BD; however, this relationship may be partly related to BMI (Zou et al., 2022a). Hence, we included BMI percentile in the primary analysis. We also included current and lifetime mania and depression scores as sensitivity analyses. Current mood was utilized as a covariate as it was more proximal to CRP measurement and our group has previously found elevated CRP levels to be associated with symptomatic youth with BD (Zou et al., 2022a). Medications, including lithium have also been associated with altered CRP levels (Queissner, Lenger, Birner, et al., 2021), and were thus also included as covariates in sensitivity analyses. Literature indicates lifetime smoking exposure is associated with elevated CRP, even following cessation (Hastie, Haw, & Pell, 2008). Thus, we included lifetime smoking as a covariate in sensitivity analyses.

### Statistical analysis

The IBM Statistical Package for Social Sciences (SPSS) version 27 was used to conduct all statistical analyses (IBM; NY, USA). Group differences in clinical and demographic characteristics between BD youth with a history of suicide attempt (BD_SA_), those with a history of NSSI (BD_NSSI_), those with no history of suicide attempt or NSSI (BD_No-SA/NSSI_), and HC youth were investigated. Normality and homogeneity of variance for continuous variables were assessed using Shapiro–Wilk’s and Levene’s test, respectively. Categorical variables were analyzed using chi-square tests and continuous variables were examined with one-way analysis of variance (ANOVA) or t-tests to compare group differences with respect to demographic and clinical characteristics. Welch’s t-test was used in circumstances where Levene’s test was violated.

The analysis of covariance (ANCOVA) test was utilized to examine group differences in CRP, with age, sex, and BMI percentile included as covariates. Normality of model residuals was examined qualitatively using a Q-Q plot. Given that the raw CRP values yielded model residuals that were not normally distributed, CRP underwent a logarithmic transformation. Log-transformed CRP values met normality assumptions for ANOVA (Supplemental Figure 1). Sensitivity analyses were conducted to examine the effects of lifetime smoking status, current MRS/DRS scores, lifetime most severe past MRS/DRS scores, current lithium, and current second-generation antipsychotic use. These variables were entered individually as covariates in a series of ANCOVA models, also controlling for age, sex, and BMI percentile. Any significant outcomes in ANCOVA or ANOVA tests were further analyzed using Tukey’s pairwise post hoc tests.

## Results

### Demographic and clinical characteristics

A total of 211 youth (23 BD_SA_, 45 BD_NSSI_, 39 BD_No-SA/NSSI_ and 104 HC) completed blood sampling and interview procedures, demographic and physical characteristics relevant to each group are reported in Table 1. BD_No-SA/NSSI_ and BD_NSSI_ groups had significantly higher mean age, lower global functioning scores for the highest level of functioning, and lower global functioning current episode scores compared to HC youth. There was a significantly greater proportion of females in the BD_NSSI_ compared to BD_No-SA/NSSI_ and HC. The BD_No-SA/NSSI_ group also had a significantly higher percentage of Caucasian participants compared to HC. BMI and BMI percentile were significantly higher in all groups with BD compared to HC.

**Table 1.** Demographic and physical characteristics

	Controls (*n* = 104)	BD_No-SA/NSSI_ (*n* = 39)	BD_NSSI_ (*n* = 45)	BD_SA_ (*n* = 23)	Test statistic	*p*-value	Effect size	
Demographics								
Age	17.3 ± 2.0	18.5 ± 1.7	18.2 ± 1.8	17.9 ± 1.7	4.89	<0.01^a,b^	0.07	
SES	4.4 ± 0.8	4.4 ± 0.9	4.2 ± 1.0	3.7 ± 1.1	3.82	0.01^c,e,f^	0.06	
Sex (*n*, % female)	54 (52)	18 (46)	38 (84)	16 (70)	17.97	<0.001^b,d^	0.29	
Race (*n*, % Caucasian)	57 (55)	26 (84)	32 (78)	13 (62)	12.75	<0.01^a^	0.25	
Intact family (*n*, %)	70 (67)	21 (70)	24 (59)	11 (52)	2.72	0.44	0.12	
Tanner stage*	4.2 ± 0.7	4.5 ± 0.6	4.7 ± 0.6	4.4 ± 0.9	3.72	0.01^b^	0.06	
Physical characteristics							
BMI (kg/m^2^)	21.8 ± 3.7	24.1 ± 3.7	24.5 ± 5.2	26.0 ± 5.6	8.80	<.001^a,b,c^	0.11	
BMI percentile	50.7 ± 28.9	65.3 ± 23.9	64.0 ± 28.1	74.2 ± 24.5	6.74	<0.001^a,b,c^	0.09	
CRP (mg/L)	0.8 ± 0.9	1.4 ± 2.3	1.8 ± 2.2	2.8 ± 3.1	6.88	<0.001^c,e^	0.09	

*Note*: Test statistic represents *F*, *t*, or χ^2^. Effect sizes are Cramer’s *V*, *η* ^2^, or Cohen’s *d.* Values are reported in mean ± standard deviation unless otherwise indicated. BD, bipolar disorder; CGAS, Children’s Global Assessment Scale; SES, socioeconomic status; SD, standard deviation. Raw CRP means and standard deviations reported. Post hoc pairwise comparisons: a = significant BD_No-SA/NSSI_ versus controls; b = significant BD_NSSI_ versus controls; c = significant BD_SA_ versus controls; d = significant BD_No-SA/NSSI_ versus BD_NSSI_; e = significant BD_SA_ versus BD_No-SA/NSSI_; f = significant BD_SA_ versus BD_NSSI_.

* Homogeneity of variance violated, Welch test reported.

Clinical characteristics of 107 youth with BD are reported by suicide risk groups in Table 2. Prevalence of lifetime psychosis was significantly greater in the BD_No-SA/NSSI_ group compared to BD_NSSI_. Higher rates of lifetime suicidal ideation and lifetime any anxiety disorder were seen in the BD_NSSI_ group compared to the BD_No-SA/NSSI_ group. There was a greater incidence of lifetime suicidal ideation, encounters with police, and lifetime psychiatric hospitalization in the BD_SA_ group compared to BD_NSSI_ and BD_No-SA/NSSI_ groups. Number of anxiety disorders, current, and lifetime depression scores were significantly greater in the BD_NSSI_ group compared to the BD_No-SA/NSSI_ group. Finally, compared to the BD_No-SA/NSSI_ group, the BD_SA_ group had significantly higher lifetime depression score, current mania score, and use of SSRI antidepressants. There were no significant differences among groups related to current medication use and family psychiatric history, including family history of suicide attempt.

**Table 2.** Clinical characteristics

	BD_No-SA/NSSI_ (*n* = 39)	BD_NSSI_ (*n* = 45)	BD_SA_ (*n* = 23)	Test statistic	*p*-value	Effect size
Clinical characteristics, *n* (%)						
BD-I	20 (51)	10 (22)	7 (30)	11.30	0.07	0.28
BD-II	8 (21)	17 (38)	9 (39)			
BD-NOS	11 (28)	18 (40)	7 (30)			
Age of onset	14.7 ± 3.3	14.7 ± 2.5	14.8 ± 1.9	0.02	0.99	<0.001
Lifetime psychosis	16 (47)	9 (21)	5 (23)	7.18	0.03^a^	0.27
Lifetime suicide attempts	0	0	23 (100)	–	–	–
Lifetime self-injurious behavior	0	45 (100)	20 (87)	–	–	–
Lifetime suicidal ideation	18 (49)	35 (78)	23 (100)	19.86	<0.001^a,b,c^	0.44
Legal history	5 (17)	9 (22)	10 (59)	10.82	<0.01^b,c^	0.35
Lifetime physical abuse	1 (3)	1 (2)	3 (18)	5.56	0.06	0.25
Lifetime sexual abuse	1 (3)	2 (5)	2 (12)	1.48	0.48	0.13
Lifetime any abuse (physical and/or sexual)	3 (10)	2 (5)	5 (28)	6.65	0.04^c^	0.27
Lifetime psychiatric hospitalization	17 (44)	18 (40)	19 (83)	12.22	<0.01^b,c^	0.34
Current depression score	10.8 ± 10.9	16.1 ± 9.4	19.9 ± 12.5	5.69	<0.01^b^	0.10
Lifetime depression score*	25.6 ± 10.2	33.9 ± 8.6	35.8 ± 10.0	11.35	<0.001^a,b^	0.18
Current mania score*	4.2 ± 6.6	8.9 ± 9.7	14.3 ± 12.1	8.72	<0.001^b^	0.14
Lifetime mania score	30.7 ± 10.0	31.8 ± 8.7	33.0 ± 8.1	0.47	0.63	0.01
CGAS: most severe past episode*	47.0 ± 11.7	44.7 ± 9.1	42.8 ± 9.1	0.27	0.76	0.02
CGAS: highest past year*	75.7 ± 11.7	70.8 ± 9.1	68.6 ± 7.4	3.49	0.04^b^	0.08
CGAS: past month*	71.1 ± 13.3	67.3 ± 9.5	61.5 ± 10.1	4.14	0.19	0.09
Lifetime comorbid diagnoses, *n* (%)						
ADHD	13 (33)	20 (44)	14 (61)	4.46	0.11	0.20
Oppositional defiant disorder	7 (18)	17 (38)	9 (39)	4.80	0.09	0.21
Conduct disorder	0	2 (4)	0	2.81	0.25	0.16
Any anxiety disorder	28 (72)	43 (96)	22 (96)	12.34	<0.01^a^	0.34
Number of anxiety disorders	1.6 ± 1.6	2.6 ± 1.3	2.2 ± 1.4	5.46	<0.01^a^	0.01
Substance use disorder	10 (26)	11 (24)	10 (44)	3.01	0.22	0.17
Lifetime cigarette smoking (yes/no)	16 (41)	22 (49)	15 (65)	3.40	0.18	0.18
Family psychiatric history, *n* (%)						
Psychosis	7 (18)	10 (22)	5 (22)	0.26	0.88	0.05
Mania/hypomania	19 (49)	20 (44)	7 (30)	0.34	0.85	0.06
Depression	23 (59)	36 (80)	16 (70)	4.41	0.11	0.20
Suicide attempt/completion	11 (28)	13 (29)	8 (35)	1.25	0.34	0.11
Anxiety	22 (56)	27 (60)	14 (61)	0.16	0.92	0.04
ADHD	15 (39)	11 (24)	9 (39)	2.41	0.30	0.15
Substance use disorder	14 (36)	23 (51)	11 (48)	2.06	0.36	0.14
Lifetime treatment, *n* (%)						
SGA	29 (74)	31 (69)	21 (91)	4.22	0.12	0.20
Lithium	14 (36)	9 (20)	6 (26)	2.69	0.26	0.16
SSRI antidepressants	12 (31)	25 (56)	17 (74)	11.58	<0.01^b^	0.33
Non-SSRI antidepressants	6 (15)	7 (16)	8 (35)	4.27	0.12	0.20
Stimulants	9 (23)	8 (18)	9 (39)	3.82	0.15	0.19
Lamotrigine	10 (26)	14 (31)	6 (26)	0.37	0.83	0.06
Any medication	35 (90)	40 (89)	23 (100)	2.71	0.26	0.16
Any psychosocial treatment	22 (61)	39 (91)	22 (96)	15.31	<0.001^a,b^	0.39
Current medications, *n* (%)						
SGA	25 (64)	24 (53)	19 (83)	5.64	0.06	0.23
Lithium	12 (31)	8 (18)	5 (22)	2.01	0.37	0.14
SSRI antidepressants	4 (10)	11 (24)	6 (26)	3.44	0.18	0.18
Non-SSRI antidepressants	1 (3)	2 (4)	2 (9)	1.23	0.54	0.11
Stimulants	3 (8)	6 (13)	3 (13)	0.77	0.68	0.09
Lamotrigine	10 (26)	11 (24)	5 (22)	0.12	0.94	0.03
Any Medication	32 (82)	37 (82)	22 (96)	2.59	0.27	0.16

*Note*: Test statistic represents *F*, *t*, or χ^2^. Effect sizes are Cramer’s *V*, *η* ^2^, or Cohen’s *d.* Values are reported in mean ± standard deviation unless otherwise indicated. BD = Bipolar disorder; ADHD = Attention Deficit-Hyperactivity Disorder; SGA = Second-generation Antipsychotic; SSRI = Selective Serotonin Reuptake Inhibitor. Depression score based on Depression Rating Scale and mania score based on Mania Rating Scale. Post hoc pairwise comparisons: a = significant BD_No-SA/NSSI_ versus BD_NSSI_; b = significant BD_SA_ versus BD_No-SA/NSSI_; c = significant BD_SA_ versus BD_NSSI_.

* Homogeneity of variance violated, Welch test reported.

### CRP and suicide risk

**Figure 1.**
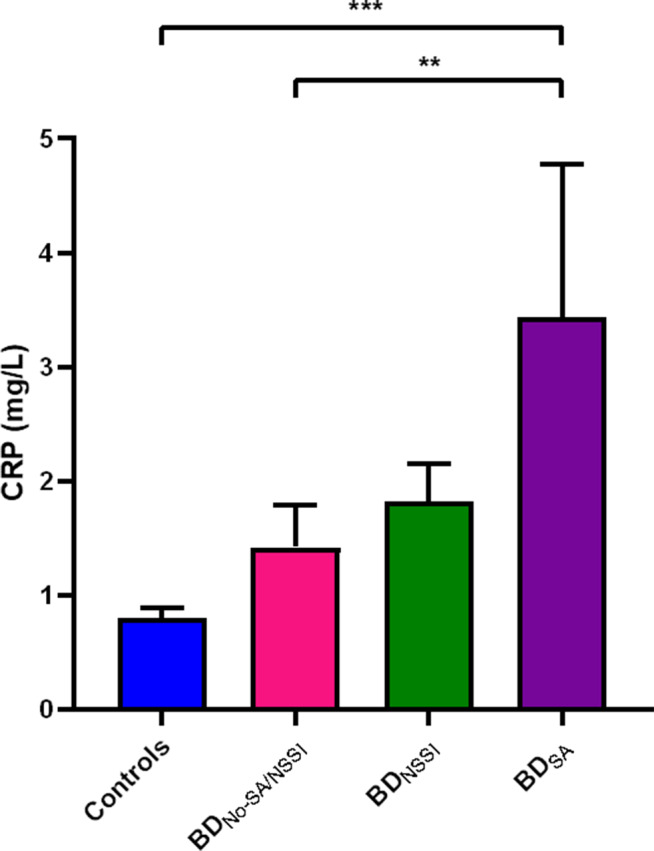
ANCOVA analysis of log-transformed mean CRP levels. Mean CRP levels were significantly different between groups (*F*_3,204_ = 3.40, *p* = 0.02, *η_p_* ^2^ = 0.05). Post hoc analyses indicated significantly higher CRP levels in the BD_SA_ group (2.83 ± 3.07 mg/L) compared with the controls (0.81 ± 0.90; *p* = 0.003) and BD_No-SA/NSSI_ (1.42 ± 2.31, *p* = 0.01) groups. The difference in CRP levels between the BD_SA_ group (2.83 ± 3.07 mg/L) and the BD_NSSI_ group (1.79 ± 2.17 mg/L; *p* = 0.11) was not significant. Groups: Controls = Healthy Controls, BD_No-SA/NSSI_ = youth with BD and history of no suicide attempt or NSSI, BD_NSSI_ = youth with BD and history of NSSI, BD_SA_ = youth with BD and history of suicide attempt.

In multivariable analyses controlling for age, sex, and BMI percentile, mean CRP levels were significantly different between groups with a medium effect size (Figure 1; *F*_3,204_ = 3.40, *p* = 0.02, *η_p_* ^2^ = 0.05). Post hoc analyses indicated significantly higher CRP levels in the BD_SA_ group (2.83 ± 3.07 mg/L) compared with the HC (0.81 ± 0.90; *p* < 0.001) and BD_No-SA/NSSI_ (1.42 ± 2.31, *p* = 0.008) groups. However, the difference in CRP levels between the BD_SA_ group (2.83 ± 3.07 mg/L) and the BD_NSSI_ group (1.79 ± 2.17 mg/L; *p* = 0.11) was not significant.

### Sensitivity analyses

The results regarding group differences in CRP levels of the BD_SA_ group compared to HC and BD_No-SA/NSSI_ groups remained significant when conducting separate sensitivity analyses for current depression score (*F*_3,203_ = 3.31, *p* = 0.02, *η_p_* ^2^ = 0.05), current mania score (*F*_3,203_ = 4.05, *p* < 0.01, *η_p_* ^2^ = 0.06), current lithium use (*F*_3,203_ = 3.58, *p* = 0.02, *η_p_* ^2^ = 0.05), current second generation antipsychotic use (*F*_3,203_ = 4.42, *p* = 0.03, *η_p_* ^2^ = 0.04), lifetime mania score (*F*_3,203_ = 4.53, *p* < 0.01, *η_p_* ^2^ = 0.06), and lifetime smoking (*F*_3,203_ = 2.71, *p* = 0.046, *η_p_* ^2^ = 0.04) as covariates in combination with age, sex, and BMI percentile. Group differences were no longer significant in sensitivity analysis, controlling for the most severe past depression score (*p* = 0.19).

## Discussion

Building on prior findings in adults with BD, this study examined CRP in relation to suicidality among youth with BD in a relatively large clinical sample. CRP levels were significantly higher among youth with BD and a history of suicide attempts compared to youth with BD with no history of non-suicidal self-injury or suicide attempt and HCs. Among youth with BD, it is noteworthy that suicide attempt was not associated with significantly different CRP levels compared to non-suicidal self-injury. These results add to the limited literature regarding the association of CRP and suicide risk, extending this line of research to youth with BD who comprise a group at highly elevated risk for suicide.

Prior research based on a largely overlapping sample found minimal differences in the clinical characteristics of BD_SA_ and BD_NSSI_. This may explain the specific association between CRP and suicide attempt in the current study. Given that the majority of BD_SA_ youth also had lifetime NSSI, one could posit that their elevated CRP levels reflect an additive effect with NSSI. However, given that CRP levels for BD_NSSI_ did not differ from BD_No-SA/NSSI_ youth or HC, it appears that the association with elevated CRP is specific to suicide attempts. A prior study of 51 adolescents found that higher IL-6 levels predict greater frequency of suicide attempts or NSSI over three months. Post hoc analyses were significant for NSSI but not suicide attempts (Bai, Asarnow, Babeva, & Irwin, 2024). A similar pattern was evident for CRP, but it was not significant (Bai et al., 2024). The discrepancy between the prior and current studies may relate to differences in psychiatric diagnoses and timeframe (lifetime versus three months).

CRP has also previously been proposed as a trait marker of suicide, as levels differed significantly between suicide attempters and non-attempters, whereas there were no differences between individuals with recent versus distant suicide attempts in a study of 600 adult inpatients with varying Axis I disorders (Courtet, Jaussent, Genty, et al., 2015). Meanwhile, a study observing inflammation in adolescent females with high risk of NSSI and suicidal behavior and history of NSSI found no association between CRP and history of NSSI in comparison to HC (Kindler et al., 2022). These findings support the current findings and the narrative that a history of suicide attempt may be marked through relatively greater CRP levels compared to groups with no history of self-harm, while the same is not seen in individuals with a history of NSSI. Events of NSSI may function to minimize negative emotions and suppress more lethal impulses of self-harm (Pompili, Goracci, Giordano, et al., 2015), potentially representing a less severe, if not less chronic/recurrent, psychopathological profile compared to a suicide attempt.

While the current cross-sectional study was not designed to evaluate temporal associations or mechanisms, prior informative studies are relevant to the current findings. For example, treatment with interferon-α, a pro-inflammatory cytokine, is known to precipitate depression among a subset of individuals (Lotrich, Albusaysi, & Ferrell, 2013). However, there is also evidence that interferon-α treatment may precipitate new-onset suicide attempts (Sockalingam, Links, & Abbey, 2011). This speaks to the potential mechanistic effect of elevated CRP on the risk of suicide attempt. Potential factors underlying the link between CRP and suicide attempt include genetics, the kynurenine pathway, neurocognitive dysfunction, monoamine metabolism, and the hypothalamic–pituitary axis (Brundin, Bryleva, & Thirtamara Rajamani, 2016). Oxidative stress, which is often associated with inflammation, has also been associated with lower white matter integrity in youth with BD compared to healthy controls (Zou et al., 2022b). Another plausible mechanism may be neuroinflammation as a result of CRP inducing greater blood–brain barrier permeability, which may be further exaggerated due to obesity (Hsuchou, Kastin, Mishra, & Pan, 2012). In a prior paper published by our group in a subset of the current sample, elevated CRP was associated with lower cortical thickness and higher cortical surface area, supporting the aforementioned hypothesis (Shao et al., 2023). Current hypotheses for inflammation in BD propose that increased levels of cell death and neuronal density in the central nervous system lead to elevated peripheral markers of apoptosis in individuals with BD (Fries, Walss-Bass, Bauer, & Teixeira, 2019). Overall, cognitive impairment associated with suicide attempts or NSSI may be explained through the neuroinflammatory effects of CRP.

Notably, we found differences in clinical characteristics and course of illness between groups of youth with BD. For instance, youth with BD and a history of suicide attempt demonstrated significantly higher SSRI antidepressant use and trended towards higher proportional use of SGAs, stimulants, and non-SSRI antidepressants compared to other youth with BD without a history of suicide attempt. Furthermore, youth with BD and a history of suicide attempt also indicated significantly greater depression and mania scores compared to youth with no suicide attempt or NSSI. This may represent a more severe course of illness, requiring increased use of medication. Significantly greater lifetime suicidal ideation and legal history in the BD_NSSI_ and BD_SA_ groups compared to BD_No-SA/NSSI_ also align with prior literature describing past suicidal ideation and impulsivity as risk factors/predictors of suicide attempt or NSSI.

There are several limitations of the current study that warrant discussion. First, the cross-sectional design of this study limits the ability to interpret directionality of the findings and limits causal inference due to potential confounding factors. Variables including, but not limited to, genetics, BMI, comorbid conditions, medications, adverse childhood experiences, and stress may be responsible for the association between CRP and suicide attempt. For instance, chronic stress, daily interpersonal stress, and living in vulnerable neighborhoods have been associated with elevated CRP in youth (Condon, 2018). Additionally, chronic stress is hypothesized to be linked with depression. (Hammen, 2015) Therefore, chronic stress is another variable that may have influenced CRP, which we could not assess in the current study. Nonetheless, CRP is ubiquitous and objective. As such, even if the association is not causal, it can still serve a pragmatic role in screening. Second, variability in the severity and timing of suicide attempt or NSSI may have impacted current findings. While the sample size was relatively large for a clinical study, it does not provide sufficient power to examine factors such as severity and frequency of suicide attempt or NSSI, or to test comprehensive covariate models. Particularly, only 23 individuals had a history of suicide attempt, the current study was underpowered to draw associations between the type of suicide attempt, frequency, and temporal proximity to CRP sample collection. Furthermore, consistent with the epidemiology of BD, the sample is highly heterogeneous in terms of demographic, clinical, and familial characteristics. Since only CRP was measured, we cannot conclude whether findings are specific to CRP or CRP reflects downstream effects of pro-inflammatory cytokines such as IL-6 (Sproston & Ashworth, 2018). Findings were robust to all sensitivity analyses, except for the lifetime most severe past depression score. This may underline the interplay between chronic stress, depression severity, suicide attempt, and inflammation. While BMI significantly differed between BD and HC groups, there were no differences among BD_No-SA/NSSI_, BD_NSSI_, and BD_SA_ groups. This finding may reflect mechanisms whereby BMI accounts for long-term reward and executive dysfunction in adolescence and BD independently, as shown in reviews and neuroimaging studies (Bora, McIntyre, & Ozerdem, 2018). Cognitive deficits have also been associated with elevated CRP levels in youth with BD and a history of suicide attempt and NSSI (Dickerson et al., 2013; Dimick, Sultan, Kennedy, et al., 2023), suggesting common pathways of inflammation related to cognition, BMI, and suicide attempt.

In conclusion, the current findings highlight increased CRP levels in youth with BD and a history of suicide attempt compared to youth with BD and lifetime NSSI, without a history of suicide attempt or NSSI, and healthy controls. Future studies should incorporate a comprehensive assessment of covariates such as chronic stress and psychiatric comorbidities to better isolate the independent and interactive role of CRP. Additionally, studies with prospective designs can provide greater insight into the temporal relationship of CRP and inflammation with individual risk of suicide attempt or NSSI. Additionally, the relationship of severity, intent, and method of suicide attempt or NSSI with CRP should be examined. By building upon this literature, we may better elucidate the connection between inflammation and suicide risk in youth with BD. Currently, the assessment of suicide risk in individuals with BD requires routine investigation through clinical interview, which may be unreliable due to its subjective nature. Furthermore, clinicians lack measures and BD-specific tools to evaluate suicide risk. From a clinical perspective, CRP is an accessible marker and may serve as an index of risk before or after a suicide attempt. Thus, while directionality cannot be determined by the current study, further research may provide insight into the use of CRP as a biomarker to objectively assist in the assessment of suicide risk in this at-risk population.

## Supporting information

10.1017/S0033291726103948.sm001 Desai et al. supplementary material Desai et al. supplementary material

## Supplementary material

The supplementary material for this article can be found at http://doi.org/10.1017/S0033291726103948.
