# Supplementary material for: Elevated C-reactive protein is associated with suicide attempts in youth with bipolar disorder
Source: Psychol Med. 2026 Apr 13;56:e105. doi: 10.1017/S0033291726103948 (PMC13079216; doi:10.1017/S0033291726103948)
Supplement: Desai et al. supplementary material [file S0033291726103948sup001.docx]

**Supplemental Materials**

**Dataset Details: Inclusion and Exclusion Criteria of Included Studies**

The following is a list of included studies from which the sample was drawn for the current dataset. Study title, inclusion, and exclusion criteria from the protocols of each study have been described.

**Study 1. Inflammatory Markers, Brain-Derived Neurotrophic Factor, and the Longitudinal Course of Adolescent Bipolar Disorder**

**Inclusion Criteria:** English-speaking males and females, 13-19 years of age, of any race/ethnicity; and either: 1) meet diagnostic criteria for BD-I, BD-II, or BD-NOS; OR 2) have no major psychiatric disorders and no family history of BD (as above). All participants will complete rigorous diagnostic interviews to ensure that they meet one of these requirements. Participants with BD must be symptomatic (as below).

**Exclusion Criteria:** 1) unable to provide informed consent (e.g. severe psychosis, developmental delay); 2) chronic inflammatory illness (e.g. rheumatoid arthritis); 3) daily anti-inflammatory medication including glucocorticoids; 4) infectious illness (e.g. colds) in the past 14 days. Psychotropic medications are permitted, and will be analyzed for potential inclusion as a covariate.

**Study 2. Oxidative Stress and Endothelial Function as Peripheral Biomarkers of Neurocognition in Adolescent Bipolar Disorder**

**Inclusion Criteria:** English-speaking males and females, 13-21 years of age, of any race/ethnicity, and either: 1) meet diagnostic criteria for BD; OR 2) have no major or recent psychiatric disorders (no lifetime mood or psychotic disorders, no recent alcohol or drug dependence in the past 3 months, and no recent anxiety disorders with the past 3 months) and no family history of BD or psychotic disorder (first and second degree relatives); OR have a parent or sibling (15 years or older) with BD (type I or II) but do not have any major or recent psychiatric disorders (mood, psychotic, or recent drug or alcohol dependence in the past 3 months, and no recent anxiety disorders in the past three months; except for MDD). All participants will complete rigorous diagnostic interviews to ensure that they meet one of these requirements.

**Exclusion Criteria:** 1) unable to provide informed consent (e.g. severe psychosis, developmental delay), 2) existing cardiac condition (e.g. conduction abnormality or congenital heart disease), auto-immune illness, or inflammatory illness, 3) currently taking anti-inflammatory, anti-platelet, anti-lipidemic, anti-hypertensive, or hypoglycemic agents including insulin or metformin, 4) infectious illness within the past 14 days.

**Study 3. Neural and Peripheral Approaches Linking Circuitry and Circulation in Adolescent Bipolar Disorder**

**Inclusion Criteria:** English-speaking, of any race/ethnicity, and 1) 13-20 years of age meeting diagnostic criteria for BD (type I, II, or NOS), OR 2) have a biological parent/sibling with BD, but do not have any major or recent psychiatric disorders (mood, psychotic, or recent drug or alcohol dependence in the past 3 months; excluding MDD, anxiety disorders, or ASD without language impairment), OR 3) 13-20 years of age with no major psychiatric disorders and no family history of BD. All participants will complete gold-standard semi-structured diagnostic interviews to ensure that they meet one of these requirements.

**Exclusion Criteria:** 1) unable to provide informed consent, 2) existing cardiac condition (e.g. conduction abnormality or congenital heart disease), auto-immune illness, or inflammatory illness, 3) currently taking anti-inflammatory, anti-lipidemic, anti-hypertensive agents 4) contraindications to magnetic resonance imaging (e.g. cardiac pacemaker or other implanted device) 5) neurological or cognitive impairment, 6) infectious illness within the past 14 days, 7) substance dependence in the past 3 months.

**Study 4. Study Examining Cognition, Retinal vessels and Emotions in Teens**

**Inclusion Criteria:** English-speaking males and females, 13-20 years of age, of any race/ethnicity, and either: 1) meet diagnostic criteria for BD (type I, II, or NOS/other specified bipolar and related disorder) or MDD; OR 2) have no major or recent psychiatric disorders (no lifetime mood or psychotic disorders, no recent alcohol or drug dependence in the past 3 months, and no family history of BD or psychotic disorder (first and second degree relatives); OR 3) have a biological parent/sibling with BD, but do not have any major or recent psychiatric disorders (mood, psychotic, or recent drug or alcohol dependence in the past 3 months; excluding MDD or anxiety disorders); OR 4) have a biological parent with CVSD and no family history of BD or psychotic disorder (first and second degree relatives), but do not have any major or recent psychiatric disorders (mood, psychotic, or recent drug or alcohol dependence in the past 3 months; excluding MDD or anxiety disorders). All participants will complete rigorous diagnostic interviews to ensure that they meet one of these requirements. In cases when the sibling or parent of possible participants in group 3 is unable to come in, and a semi-structured diagnostic interview cannot be completed with another informant, we will receive consent from the individual with bipolar disorder to access their medical records (via a Release of Information form) in order to confirm their diagnosis of bipolar disorder.

**Exclusion Criteria:** 1) unable to provide informed consent (e.g. severe psychosis, developmental delay), 2) neurological or cognitive impairments, 3) infectious illness within the past 14 days, 4) substance dependence within the past 3 months.

**Table S1.** Measurement scale for level of intent, medical threat of suicidal behaviors and PSR rating scale for suicidal ideation.

| **Intent** | |
| --- | --- |
| 0 | No information |
| 1 | Obviously no intent |
| 2 | Only minimal intent |
| 3 | Definite but still ambivalent |
| 4 | Serious |
| 5 | Very serious |
| 6 | Extreme (e.g., careful planning and every expectation of death) |
| **Medical Threat** | |
| 0 | No information |
| 1 | No danger (e.g. no effect – held pills in hand) |
| 2 | Minimal (e.g. scratch on wrist) |
| 3 | Mild (e.g. took ten aspirins – mild gastritis) |
| 4 | Moderate (e.g. took ten secobarbital sodium – briefly unconscious) |
| 5 | Severe (e.g. cut throat) |
| 6 | Extreme (e.g. respiratory arrest or prolonged coma) |
| 7 | Death |

**Supplemental Figure 1.** Q-Q plot displaying residuals for log transformed CRP values.
